# The association of class II HLA alleles with tuberculosis-associated immune reconstitution inflammatory syndrome

**DOI:** 10.1371/journal.ppat.1013497

**Published:** 2025-09-19

**Authors:** Phuti Choshi, Sarah Pedretti, Tafadzwa Chimbetete, Rama Gangula, Muki Shey, Cari Stek, Rachel P. J. Lai, Robert Wilkinson, Graeme Meintjes, Elizabeth Phillips, Jonny Peter

**Affiliations:** 1 Department of Medicine, Division of Allergy and Clinical Immunology, Groote Schuur Hospital, University of Cape Town, Cape Town, South Africa; 2 Allergy and Immunology Unit, University of Cape Town Lung Institute, Cape Town, South Africa; 3 Department of Medicine, Center for Drug Safety and Immunology, Vanderbilt University Medical Center, Nashville, Tennessee, United States of America; 4 Wellcome Centre for Infectious Diseases Research in Africa, Institute of Infectious Disease and Molecular Medicine, University of Cape Town, Cape Town, South Africa; 5 Department of Infectious Diseases, Imperial College London, London, United Kingdom; 6 The Francis Crick Institute, London, United Kingdom; 7 Department of Medicine, Division of Infectious Diseases and HIV Medicine, Groote Schuur Hospital, University of Cape Town, Cape Town, South Africa; 8 Faculty of Medicine and Dentistry, Blizard Institute, Queen Mary University of London, London, United Kingdom; 9 Institute for Immunology and Infectious Diseases, Murdoch University, Murdoch, Western Australia, Australia; National Institutes of Health-NIAID, UNITED STATES OF AMERICA

## Abstract

Genetic associations within the human leukocyte antigen (HLA) gene complex and linked genes in TB-IRIS outcomes remains population specific and not well understood. Here, we conducted a study including well characterised HIV-TB coinfected patients with (n = 86) and without (n = 124) TB-IRIS from the randomized, double-blind, prophylactic prednisone trial (PredART study) with HLA, ERAP and KIR genotyping data. We confirmed the association of TB-IRIS with lower CD4 counts pre-ART initiation. We identified nine classical class I and II HLA alleles protective against TB-IRIS, while four alleles were linked to increased risk. Associations ranged from strongly protective (HLA-DQB1*05:01, OR: 0.07, 95%CI: 0.02-0.28, Pc < 0.001) to strongly risk associated (notably DRB1*01:02, OR: 5.92, 95%CI: 1.36-26.7, Pc = 0.028), with conflicting signals at the HLA-DRB1 locus. Conditional regression analysis revealed that residue E71 at the polymorphic position 71 within the HLA-DRB1 peptide-binding groove was critical, and grouping of HLA-DRB1 alleles by the residue at position 71 corresponded with differential TB-IRIS association. In conclusion, this study identifies population-specific genetic factors influencing TB-IRIS susceptibility and highlights a potential mechanistic role for specific HLA-DRB1 residues in modulating immune responses during ART.

## Author summary

When people living with HIV who also have tuberculosis (TB) start HIV treatment, some develop a dangerous complication called tuberculosis-associated immune reconstitution inflammatory syndrome (TB-IRIS). This happens because the recovering immune system reacts too strongly against TB bacteria. Not everyone with HIV and TB develops TB-IRIS, and we don’t fully understand why. In this study, we looked at genetic differences in immune system genes among patients from a PredART trial in South Africa. We found that certain genetic markers in the HLA region, which helps the immune system recognize infections, were linked to either higher or lower risk of TB-IRIS. In particular, one small change in the structure of the HLA-DRB1 gene seemed especially important. Our findings suggest that a person’s genetic make-up influences whether they develop TB-IRIS and helps explain why this condition happens in some people but not others.

## Introduction

In persons living with human immunodeficiency virus (PLWH), tuberculosis (TB)-associated immune reconstitution inflammatory syndrome (TB-IRIS) is characterized by the recurrence, worsening or emergence of inflammatory signs and symptoms of TB in those receiving anti-TB treatment, shortly after initiating antiretroviral therapy (ART) [1]. Common manifestations of TB-IRIS include enlarged and necrotic lymph nodes, exacerbation of existing or new pulmonary lesions, abdominal pain, and neurological inflammation [2]. Known risk factors for TB-IRIS include a short interval between the initiation of anti-TB treatment and ART, low CD4 + T cell count nadir (<50 cells/μL) and extrapulmonary TB [3]. It has been proposed that the phenotype and function of TB-specific CD4 + T cells restored following ART initiation influence susceptibility to TB-IRIS [4,5]. Another complementary model suggests that TB-IRIS may also involve a hyperresponsive innate immune system with excessive macrophage activation [6], indicating that both adaptive and innate immune mechanisms, and cross-talk between active T-cells/macrophages likely contribute to pathogenesis.

The immunogenetic variations within the human leukocyte antigen (HLA) gene complex have been recognised as significant risk factors and drivers of adaptive immune responses to various infections including TB, HIV and autoimmune diseases [7,8]. Epistatically linked genes such as endoplasmic reticulum aminopeptidases (ERAP), which affect peptide trimming, and killer cell immunoglobulin-like receptors (KIR), which regulate antiviral responses mediated by natural killer (NK) cells have also been shown to be important. However, the influence of HLA in the context of the inflammatory TB-IRIS is understudied. Cytomegalovirus (CMV)-IRIS has been associated with HLA-B44 and an ancestral haplotype HLA-A2, B44, DR4 [9]. While the regulatory cytokine gene polymorphisms (TNF-α-308*2 and IL-6–174*G), linked to low cytokine production, have been shown to be protective against herpes virus-IRIS and TB-IRIS [10]. Additionally, a recent Brazilian study linked HLA-B*41, KIR2DS2, and KIR + HLA-C to IRIS onset among HIV-TB co-infected individuals [11]. However, these associations may not be generalisable to broader populations, given HLA-genetic and environmental variability across different regions. In this study, we aimed to identify genetic associations between HLA alleles, ERAP variants, and KIR genotypes and TB-IRIS outcomes, and to investigate potential interactions among these factors.

## Results

**Fig 1:**
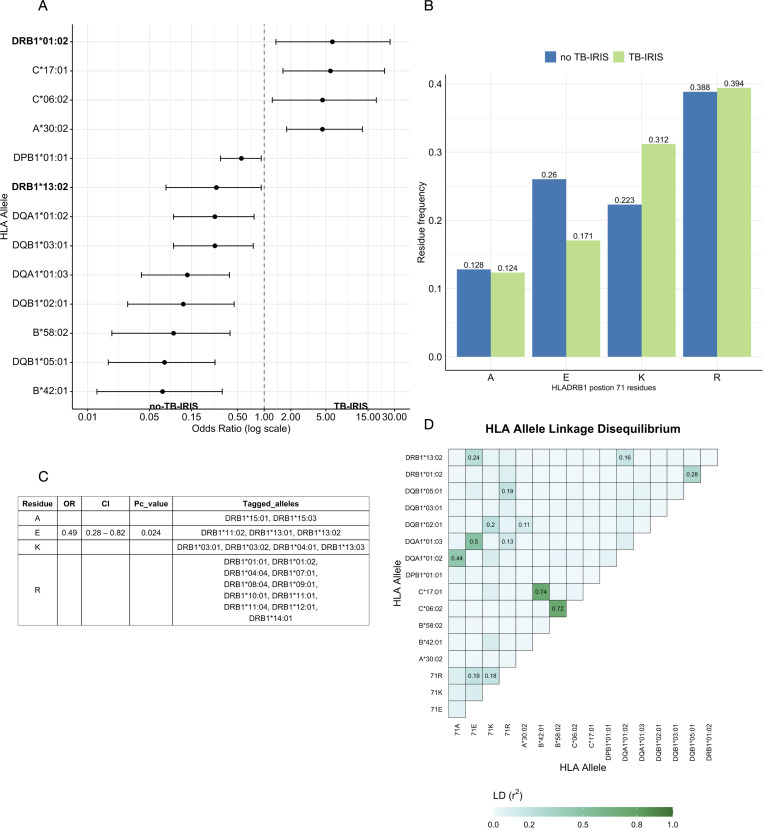
Genetic associations with TB-IRIS. A. Significantly associated risk and protective HLA alleles identified by stepwise logistic regression analysis including allele dosage. Data are presented as odds ratio (log scale) and 95% confidence interval. B. Allele frequencies of four residues at position 71 of HLA-DRB1 in the two groups. C. The association of HLA-DRB1-position 71 amino acids residues with TB-IRIS outcomes. HLA class II variants tagged by the four residues at position 71 are noted in the last column. D. Correlation coefficient plot of linkage disequilibrium between 16 associated single alleles and residues at position 71 of HLA-DRB1. Pc- FDR corrected p-value. Amino acid residues: A – alanine, E – glutamic acid, K – lysine, R – arginine. OR – odds ratio. LD – linkage disequilibrium.

We stratified participants (all PLWH) according to development of TB-IRIS versus no-TB-IRIS (S1 Table) and confirmed association (P < 0.001) of TB-IRIS with CD4 count pre-ART initiation (S1 Fig) [3]. Characteristics of participants are outlined here [1] and in S1 Table. We proceeded to identify HLA associations with TB-IRIS by conducting stepwise logistic regression analysis including 149 classic alleles across 7 HLA genes (HLA-A, B, C, DPB1, DQA1, DQB1, DRB1), adjusting for potential confounding factors (CD4 count, age, sex and prednisone exposure as a trial intervention). After false discovery rate (FDR)-correction for multiple comparisons, HLA-B*42:01, B*58:02, DPB1*01:01, DQA1*01:02, DQA1*01:03, DQB1*02:01, DQB1*03:01, DQB1*05:01 and DRB1*13:02 were found to be protective against the development of TB-IRIS. HLA-A*30:02, C*06:02, C*17:01 and DRB1*01:02 were significantly associated with TB-IRIS (Fig 1A and S2 Table).

The association signal observed at HLA-DRB1 includes both risk (DRB1*01:02) and protective (DRB1*13:02) alleles for TB-IRIS, suggesting that this locus harbours functionally relevant variation that modulates the direction of association. To specifically examine how HLA-DRB1 molecules might be interacting with antigens and T cells as part of increased risk or protection from TB-IRIS, we tested the independent effects of amino acids within polymorphic positions of HLA-DRB1 [12]. Four residues (A - alanine, E – glutamic acid, K - lysine, R - arginine) at amino acid position 71 of HLA-DR, E71 and K71 showed differences in frequency between TB-IRIS and no-TB-IRIS (Fig 1B) – only E71 showed significant protective effect (OR 0.49 95% CI 0.28 – 0.82 P = 0.008, Pc = 0.024) (Fig 1C). Residue E71 tagged one of the protective alleles DRB1*13:02. In conditional logistic regression model including position 71 residues and significantly associated HLA alleles as covariates, the protective effect of E71, B*58:02, DPB1*01:01, DQA1*01:02, DQA1*01:03, DQB1*02:01, DQB1*03:01 and DRB1*13:02 was eliminated. The effect of TB-IRIS associated alleles (C*06:02, C*17:01 and DRB1*01:02) was also eliminated after correction for multiple comparisons (S3 Table).

Conditioning for E71 explained a substantial portion of protective HLA associations. We next looked at correlation using the r^2^ measure [13] between associated alleles and residues at position 71 and found the weakened effect of DQA1*01:02, DQA1*01:03, DQB1*02:01, DRB1*13:02 was partially driven by linkage disequilibrium (r^2^ ≥ 0.2) with residues at position 71. Additionally, highly correlated or haplotypes in class I region were identified among significantly associated alleles – HLA-B*42:01, C*17:01; HLA-B*58:02, C*06:02 (Fig 1D). Analyses of ERAP 1 and 2 loci (S4 Table) and KIR genes (S5 Table) showed no significant associations with TB-IRIS after correction for multiple comparisons. Further, interaction analysis with significantly associated HLA alleles did not reveal any epistatic association with TB-IRIS outcome (S6 Table).

## Discussion

This report presents an immunogenetic analysis of susceptibility to TB-IRIS in PLWH initiating ART. By integrating classical HLA typing, amino acid level analysis, and evaluation of ERAP and KIR genes, we uncovered multiple layers of host genetic contribution to TB-IRIS risk. Consistent with previous studies implicating HLA in immune-mediated conditions, we identified several class I and class II HLA alleles significantly associated with TB-IRIS outcome. They ranged in effect from strongly protective (notably HLA-DQB1*05:01, OR: 0.07, 95%CI: 0.02-0.28, Pc < 0.001) to strongly risk associated (notably DRB1*01:02, OR: 5.92, 95%CI: 1.36-26.7, Pc = 0.028).

The role of risk and/or protective alleles in this study and those previously reported [9,14] in the functional pathophysiology of TB-IRIS is unknown. Many of the alleles protective of TB-IRIS in our cohort also seem to be risk alleles for active TB or more severe TB (S7 Table) and lower magnitude Mycobacterium TB (MTB)-specific CD4 + T-cell responses [15–18]. In these studies alleles such as HLA-B*58:02, DRB1*13:02, DQB1*0301 showed higher susceptibility to active TB. Lower magnitude T-cell responses could be hypothesised to result in less excess cytokine production and/or macrophage crosstalk thereby being protective for TB-IRIS. In contrast to this hypothesis, a large study of HIV-TB coinfected patients found HLA-A*30:02, C*17:01 and C*06:02 were high risk alleles for developing active TB disease [18], and these were associated with increased likelihood of TB-IRIS in our cohort.

The apposing risk for TB-IRIS at the well characterised DRB1 loci allowed further insights. Amino acid analysis of polymorphic positions in HLA-DRB1 revealed the effect of position 71, with the lead residue E71conferring a significant protective effect against TB-IRIS. Residue E71 in third hypervariable region of the HLA-DRB1 chain lies within the peptide binding groove of the HLA-DR molecule, and its negative charge may selectively alter peptide binding or T cell recognition, leading to critical modulation of pathogen specific CD4 + inflammatory responses with significant impacts on the development of TB-IRIS. The critical role of this polymorphic position has been demonstrated in rheumatoid arthritis, with apposing effects of K and R amino acids, with different charges, influence peptide binding [19,20]. Importantly, conditioning for E71 eliminated the associations of multiple class II alleles, including DRB1*13:02, DRB1*01:02, DQA*01:02 and DQB1*02:01 and more, indicating that E71 mediated a shared functional mechanism of protection across these alleles (S3 Table). Arginine 71 groups HLA-DRB1*01:02 (TB-IRIS risk allele in our cohort) with DRB1*04:04, both of which are established rheumatoid arthritis associated alleles (as noted in S7 Table). These alleles contain the shared epitope (SE) motif, a conserved amino acid sequence spanning positions 70–74 of the HLA-DRβ-chain. The SE enhances their ability to stably bind citrullinated peptides activating autoreactive CD4 + T cells, a key mechanism in rheumatoid arthritis pathogenesis [21,22]. Given the role of residues at 71 in the peptide-binding groove shaping HLA genes associated with TB-IRIS outcomes, it is plausible that these autoreactive CD4 + T-cells reactive to citrullinated peptides could contribute to the excess inflammatory processes associated with TB-IRIS.

In summary, these findings highlight the potential overlap between adaptive HLA-dependent T-cell responses associated with TB disease susceptibility with the development of TB-IRIS. In addition, we propose that HLA-DRB1 alleles with polymorphisms at position 71, and known associations with autoimmunity, may shape a distinct pro-inflammatory peptide repertoire compared to alleles with glutamic acid (E71) at this position, which may confer protection. Mechanistically, these differences could alter the hydrophobicity and structural properties of the peptide-binding groove, modulating epitope specificity and T cell activation thresholds during TB infection and immune reconstitution.

## Materials and methods

### Ethics

Participants included in this study participated in the randomized, double-blind, placebo-controlled trial (PredART) to assess whether prophylactic prednisone can safely reduce the incidence of TB-IRIS in patients at high risk [1]. The randomised trial and inclusion of participants in this study was approved by the University of Cape Town Human Research Ethics Committee (Ref#136/2013 and Ref#R031/2018), including the generation of genomic data and association with phenotypes. All participants gave their written informed consent.

### Study participants and genotyping

A total of 240 patients of uniform ethnicity were enrolled in the randomised trial. Trial design, participants characteristics and trial endpoints are fully described here [1]. A total of 210 patients had enough DNA for genotyping in the current study. The demographics summary of participants included are summarised in S1 Table. On enrolment all participants were PLWH initiating ART (and had not previously received ART), had started TB treatment within 30 days before initiating ART, and had a CD4 count of 100 cells or fewer per microliter. During the trial, n = 86/210 developed TB-IRIS within 12 weeks of initiating ART and n = 124/210 did not. Their CD4 counts were measured again at week 12 (S1 Table).

### Genotyping and data analysis

HLA, KIR and ERAP sequencing were performed as previously described [23,24] and summarized in supplementary materials and methods (S1 Text). **HLA association analysis** - to quantify the association between HLA alleles and TB-IRIS outcome, we used a stepwise logistic regression model with each allele of interest as the independent variable (number of copies: 0, 1, 2) and TB-IRIS outcome as the dependent variable. The model for the primary analysis was adjusted for potential confounding factors age, sex and CD4 count. The significance of HLA alleles in the logistic regression model was tested using the Wald test while accounting for multiple comparisons using Benjamini-Hochberg procedure to control the false discovery rate (FDR). Only alleles with FDR-correct P value <0.05 were considered significant and subsequently analysed. A post-hoc analysis was conducted separately to assess potential gene-to-gene interaction effects of HLA alleles with epistatically linked genes, ERAP and KIR. **HLA haplotype frequency estimation and assessment of departure from HWE** - we applied an expectation–maximization algorithm approach and the chi-square test implemented in BIGDAWG (v.3.0.3). We limited typing results to the first field of the nomenclature for this analysis. We reported P values obtained from the exact Chi-square test on each of the eight HLA loci (HLA-A, -B, -C, -DPB1, -DQA1, -DQB1 and -DRB1) in the cases and controls separately.

## Supporting information

S1 Fig CD4 count pre-ART initiation. Standard boxplots show median and interquartile range of CD4 count measurements for participants with HIV/TB co-infection pre-antiretroviral therapy (ART). From the PredART trial *n = 124* participants with no TB-IRIS and *n = 86* with TB-IRIS were included in this study. CD4 count was measured again at week 12 (median and interquartile range are indicated in S1 Table). T-test determined *P* value shown top of the graph. *P* value <0.05 considered significant.
(PDF)

S1 Table Demographics summary of PredART study participants. IQR – interquartile range. PredART – prednisone antiretroviral therapy.
(PDF)

S2 Table Stepwise conditional logistic regression analysis including 149 classic alleles across 7 HLA genes. OR – odds ratio. CI – confidence interval. P-adjust – FDR corrected p-value. HLA – human leukocyte antigen.
(PDF)

S3 Table Logistic regression analysis conditioning for HLA-DRB1 position 71 residues. OR – odds ratio. CI – confidence interval. P-adjust – FDR corrected p-value. HLA – human leukocyte antigen.
(PDF)

S4 Table Effect of ERAP1 and 2 SNPs on TBIRIS outcome. OR – odds ratio. CI – confidence interval. P-adjust – FDR corrected p-value. ERAP – endoplasmic reticulum aminopeptidase. SNP – single nucleotide polymorphism.
(PDF)

S5 Table Genetic association between KIR genes TBIRIS outcome. OR – odds ratio. CI – confidence interval. P-adjust – FDR corrected p-value. KIR – killer immunoglobulin receptor.
(PDF)

S6 Table Interactions between leading HLA alleles and ERAP1 SNPs. OR – odds ratio. CI – confidence interval. P-adjust – FDR corrected p-value. HLA – human leukocyte antigen. ERAP – endoplasmic reticulum aminopeptidase. SNP – single nucleotide polymorphism.
(PDF)

S7 Table HLA alleles significantly associated with TB-IRIS outcome in our cohort and their previous associations with tuberculosis and autoimmune diseases. OR – odds ratio. CI – confidence interval. P-adjust – FDR corrected p-value. HLA – human leukocyte antigen. HLA – human leukocyte antigen. TB- tuberculosis.
(PDF)

S1 Data Genotype data presented in the manuscript. (TXT)

S1 Text Supplementary methods and materials. (PDF)
