# Supplementary figures and images for: The association of class II HLA alleles with tuberculosis-associated immune reconstitution inflammatory syndrome
Source: PLoS Pathog. 2025 Sep 19;21(9):e1013497. doi: 10.1371/journal.ppat.1013497 (PMC12510654; doi:10.1371/journal.ppat.1013497)

**S1 Fig. CD4 count pre-ART initiation**

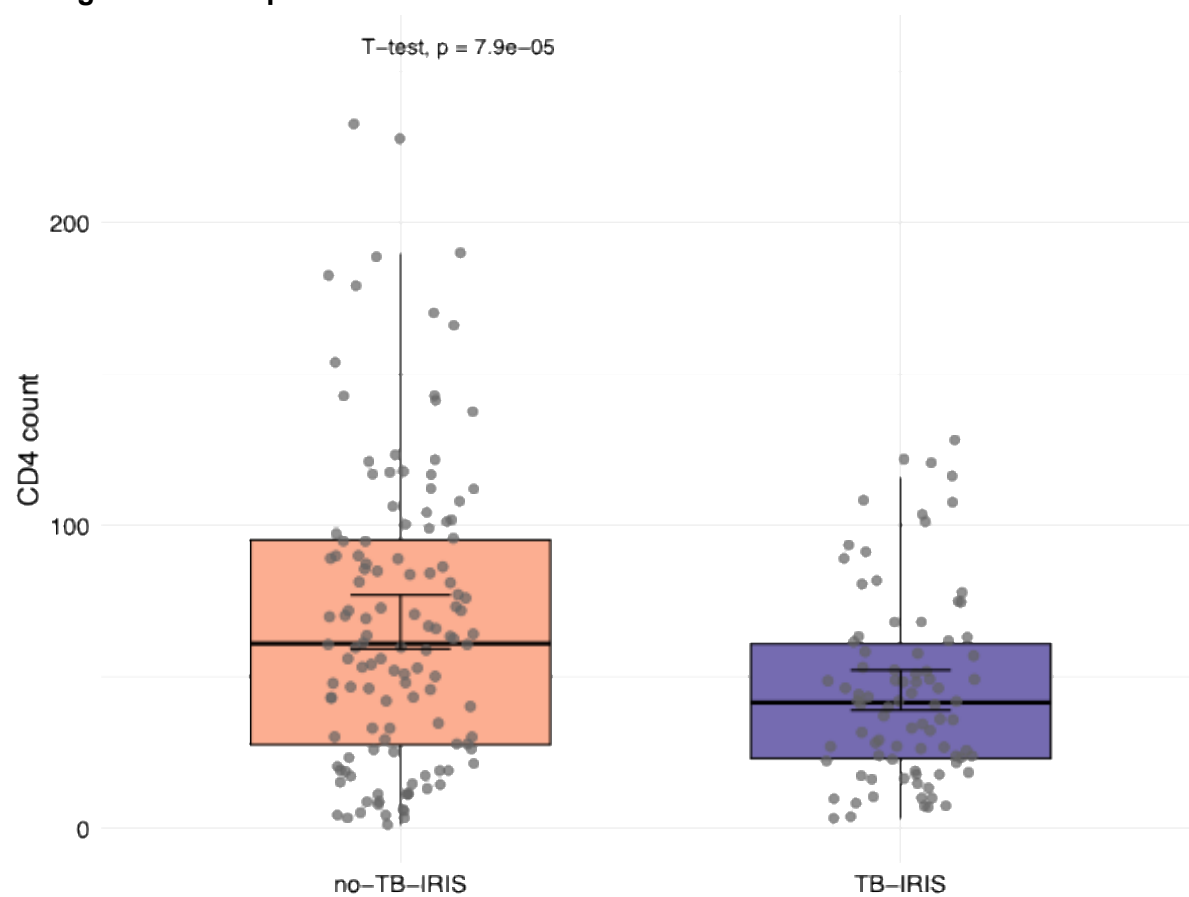

Supplement: S1 Fig — Standard boxplots show median and interquartile range of CD4 count measurements for participants with HIV/TB co-infection pre-antiretroviral therapy (ART). From the PredART trial n = 124 participants with no TB-IRIS and n = 86 with TB-IRIS were included in this study. CD4 count was measured again at week 12 (median and interquartile range are indicated in S1 Table). T-test determined P value shown top of the graph. P value <0.05 considered significant. (PDF) [file ppat.1013497.s001.pdf]
