# Supplementary material for: The association of class II HLA alleles with tuberculosis-associated immune reconstitution inflammatory syndrome
Source: PLoS Pathog. 2025 Sep 19;21(9):e1013497. doi: 10.1371/journal.ppat.1013497 (PMC12510654; doi:10.1371/journal.ppat.1013497)
Supplement: S1 Table — IQR – interquartile range. PredART – prednisone antiretroviral therapy. (PDF) [file ppat.1013497.s002.pdf]

S1 Table. Demographics summary of PredART study participants

**Characteristic**	**no-TB-IRIS n=124**	**TB-IRIS n=86**
Age at baseline (IQR)	39 (33-45)	35 (29-40)
Male	75 (60%)	53 (62%)
HIV	124 (100%)	86 (100%)
HIV viral load at baseline	316824 (122105-677359)	387359 (207290-927953)
CD4 count at baseline	61 (28-95)	42 (23-61)
Prednisone exposure as trial intervention	70 (56%)	35 (41%)
CD4 count at week 12	154 (106-227)	145 (89-212)
