# Supplementary material for: The association of class II HLA alleles with tuberculosis-associated immune reconstitution inflammatory syndrome
Source: PLoS Pathog. 2025 Sep 19;21(9):e1013497. doi: 10.1371/journal.ppat.1013497 (PMC12510654; doi:10.1371/journal.ppat.1013497)
Supplement: S2 Table — OR – odds ratio. CI – confidence interval. P-adjust – FDR corrected p-value. HLA – human leukocyte antigen. (PDF) [file ppat.1013497.s003.pdf]

S2 Table. Stepwise conditional logistic regression analysis including 149 classic alleles across 7 HLA genes

Allele	OR	95% CI-lower	95% CI-upper	p-adjust	TB-IRIS frequency	No-TBIRIS frequency
A*3002	4.57	1.80	12.94	0.0034	0.0872	0.0484
B*4201	0.07	0.01	0.33	0.0026	0.0988	0.0968
B*5802	0.09	0.02	0.41	0.0034	0.0872	0.1290
C*0602	4.58	1.23	18.63	0.0316	0.1395	0.1653
C*1701	5.63	1.63	23.11	0.0129	0.1453	0.1089
DPB1*0101	0.55	0.32	0.92	0.0316	0.3023	0.3468
DQA1*0102	0.28	0.09	0.77	0.0221	0.2500	0.2702
DQA1*0103	0.13	0.04	0.40	0.0010	0.1105	0.1452
DQB1*0201	0.12	0.03	0.46	0.0034	0.0698	0.0685
DQB1*0301	0.28	0.09	0.75	0.0203	0.1395	0.1250
DQB1*0501	0.07	0.02	0.28	0.0003	0.0872	0.1371
DRB1*0102	5.92	1.36	26.67	0.0284	0.0349	0.0403
DRB1*1302	0.29	0.08	0.93	0.0452	0.0291	0.0806
