# Supplementary material for: The association of class II HLA alleles with tuberculosis-associated immune reconstitution inflammatory syndrome
Source: PLoS Pathog. 2025 Sep 19;21(9):e1013497. doi: 10.1371/journal.ppat.1013497 (PMC12510654; doi:10.1371/journal.ppat.1013497)
Supplement: S3 Table — OR – odds ratio. CI – confidence interval. P-adjust – FDR corrected p-value. HLA – human leukocyte antigen. (PDF) [file ppat.1013497.s004.pdf]

S3 Table. Logistic regression analysis conditioning for HLA-DRB1 position 71 residues

**Allele/Residue**	**OR**	**95% CI-lower**	**95% CI-upper**	**p-adjust**	**TB-IRIS frequency**	**No-TBIRIS frequency**
A*3002	4.90	1.92	13.70	**0.017**	0.0872	0.0484
B*4201	0.10	0.02	0.44	**0.045**	0.0988	0.0968
B*5802	0.10	0.02	0.47	0.059	0.0872	0.1290
C*0602	5.14	1.33	22.07	0.365	0.1395	0.1653
C*1701	5.83	1.70	23.48	0.110	0.1453	0.1089
DPB1*0101	0.49	0.27	0.84	0.185	0.3023	0.3468
DQA1*0102	1.19	0.40	3.58	1.000	0.2500	0.2702
DQA1*0103	0.91	0.23	3.79	1.000	0.1105	0.1452
DQB1*0201	0.23	0.07	0.69	0.178	0.0698	0.0685
DQB1*0301	0.79	0.34	1.81	1.000	0.1395	0.1250
DQB1*0501	0.17	0.05	0.49	**0.015**	0.0872	0.1371
DRB1*0102	3.91	0.90	16.90	1.000	0.0349	0.0403
DRB1*1302	0.62	0.09	3.39	1.000	0.0291	0.0806
**HLA-DR residue P71**						
A71	0.06	0.00	0.72	0.514	0.1235	0.1281
E71	0.04	0.00	0.66	0.473	0.1706	0.2603
K71	0.24	0.01	2.65	1.000	0.3118	0.2231
R71	0.15	0.01	1.44	1.000	0.3941	0.3884
