# Supplementary material for: The association of class II HLA alleles with tuberculosis-associated immune reconstitution inflammatory syndrome
Source: PLoS Pathog. 2025 Sep 19;21(9):e1013497. doi: 10.1371/journal.ppat.1013497 (PMC12510654; doi:10.1371/journal.ppat.1013497)
Supplement: S4 Table — OR – odds ratio. CI – confidence interval. P-adjust – FDR corrected p-value. ERAP – endoplasmic reticulum aminopeptidase. SNP – single nucleotide polymorphism. (PDF) [file ppat.1013497.s005.pdf]

S4 Table. Effect of ERAP1 and 2 SNPs on TBIRIS outcome

Loci	SNP	OR	95% CI-lower	95% CI-upper	p-value	p-adjust
ERAP1	rs3734016	2.94	0.79	13.60	0.111	0.209
	rs73148308	0.08	0.002	1.20	0.069	0.192
	rs26653	0.37	0.15	0.85	**0.019**	0.103
	rs26618	0.39	0.09	1.60	0.192	0.326
	rs27895	1.43	0.45	4.61	0.539	0.704
	rs2287987	0.57	0.12	2.63	0.471	0.704
	rs27434	0.43	0.003	6.56	0.584	0.705
	rs73144471	25.83	1.48	983.20	**0.024**	0.103
	rs27529	8.79	0.68	1261.93	0.103	0.209
	rs17482078	0.97	0.26	3.60	0.962	0.964
	rs27044	2.14	0.92	5.28	0.079	0.192
ERAP2	rs2549782	0.83	0.49	1.42	0.501	0.704
