# Supplementary material for: The association of class II HLA alleles with tuberculosis-associated immune reconstitution inflammatory syndrome
Source: PLoS Pathog. 2025 Sep 19;21(9):e1013497. doi: 10.1371/journal.ppat.1013497 (PMC12510654; doi:10.1371/journal.ppat.1013497)
Supplement: S5 Table — OR – odds ratio. CI – confidence interval. P-adjust – FDR corrected p-value. KIR – killer immunoglobulin receptor. (PDF) [file ppat.1013497.s006.pdf]

S5 Table. Genetic association between KIR genes TBIRIS outcome

KIR Gene	OR	95% CI-lower	95% CI-upper	p-value	p-adjust
KIR2DL1	1.20	0.08	19.89	0.888	0.992
KIR2DL2	0.23	0.01	1.80	0.164	0.600
KIR2DL3	1.41	0.19	53.86	0.743	0.924
KIR2DL5	1.48	0.55	4.02	0.437	0.913
KIR2DP1	1.49	0.35	6.57	0.583	0.913
KIR2DS1	1.48	0.25	10.60	0.664	0.913
KIR2DS2	2.76	0.70	12.46	0.149	0.600
KIR2DS3	0.72	0.27	1.89	0.500	0.913
KIR2DS4	9.53	0.12	2962.03	0.320	0.880
KIR2DS5	0.85	0.30	2.39	0.756	0.924
KIR3DL1	0.79	0.01	69.42	0.916	0.992
KIR3DL2	0.39	0.001	25.43	0.638	0.913
KIR3DL3	1.09	0.003	60.31	0.966	0.992
KIR3DP1	0.19	0.02	1.65	0.131	0.600
KIR3DS1	0.65	0.11	3.72	0.633	0.913
Cen haplotypes	1.01	0.14	36.80	0.992	0.992
Tel haplotypes	0.51	0.06	4.04	0.522	0.913
Standard haplotype	3.05	0.35	32.15	0.312	0.880
