# Supplementary material for: The association of class II HLA alleles with tuberculosis-associated immune reconstitution inflammatory syndrome
Source: PLoS Pathog. 2025 Sep 19;21(9):e1013497. doi: 10.1371/journal.ppat.1013497 (PMC12510654; doi:10.1371/journal.ppat.1013497)
Supplement: S6 Table — OR – odds ratio. CI – confidence interval. P-adjust – FDR corrected p-value. HLA – human leukocyte antigen. ERAP – endoplasmic reticulum aminopeptidase. SNP – single nucleotide polymorphism. (PDF) [file ppat.1013497.s007.pdf]

S6 Table. Interactions between leading HLA alleles and ERAP1 SNPs

ERAP1 SNP:HLA combination	OR	95% CI-lower	95% CI-upper	p-value	p-adjust
rs26653:A*3002	1.36	0.75	2.54	0.313	0.313
rs26653:C*0602	0.95	0.64	1.38	0.782	0.782
rs26653:C*1701	1.08	0.73	1.60	0.703	0.703
rs26653:DPB1*0101	0.75	0.54	1.01	**0.060**	0.120
rs26653:DQA1*0102	1.07	0.79	1.47	0.653	0.653
rs26653:DQA1*0103	0.88	0.52	1.46	0.631	0.631
rs26653:DQB1*0201	1.19	0.71	2.03	0.504	0.504
rs26653:DQB1*0301	0.95	0.62	1.42	0.817	0.817
rs26653:DQB1*0501	0.82	0.49	1.34	0.439	0.439
rs26653:DRB1*0102	0.71	0.24	1.70	0.453	0.453
rs26653:DRB1*1302	0.58	0.25	1.13	0.113	0.227
rs73144471:A*3002	1.31	0.87	2.01	0.198	0.198
rs73144471:C*0602	0.89	0.64	1.23	0.485	0.485
rs73144471:C*1701	1.12	0.82	1.55	0.472	0.472
rs73144471:DPB1*0101	0.89	0.71	1.10	0.282	0.565
rs73144471:DQA1*0102	0.91	0.69	1.19	0.498	0.498
rs73144471:DQA1*0103	0.98	0.68	1.39	0.891	0.891
rs73144471:DQB1*0201	1.11	0.75	1.66	0.586	0.586
rs73144471:DQB1*0301	1.01	0.73	1.39	0.933	0.933
rs73144471:DQB1*0501	0.77	0.52	1.09	0.141	0.283
rs73144471:DRB1*0102	0.92	0.50	1.60	0.779	0.779
rs73144471:DRB1*1302	0.58	0.32	0.99	**0.044**	**0.088**
