# Supplementary material for: The association of class II HLA alleles with tuberculosis-associated immune reconstitution inflammatory syndrome
Source: PLoS Pathog. 2025 Sep 19;21(9):e1013497. doi: 10.1371/journal.ppat.1013497 (PMC12510654; doi:10.1371/journal.ppat.1013497)
Supplement: S7 Table — OR – odds ratio. CI – confidence interval. P-adjust – FDR corrected p-value. HLA – human leukocyte antigen. HLA – human leukocyte antigen. TB- tuberculosis. (PDF) [file ppat.1013497.s008.pdf]

S7 Table. HLA alleles significantly associated with TB-IRIS outcome in our cohort and their previous associations with tuberculosis and autoimmune diseases.

Variable	OR	p-adjust	TB	References [TB]	Autoimmunity	References [Autoimmunity]
A*3002	4.57	0.0034	MTB peptide binding	[1]	-	-
B*4201	0.07	0.0026	-	-	-	-
B*5802	0.09	0.0034	susceptibility to active TB with Quebec strain	[2]	-	-
C*0602	4.58	0.0316	susceptibility to active TB	[3]	-	-
C*1701	5.63	0.0129	susceptibility to active TB	[3]	-	-
DPB1*0101	0.55	0.0316	-	-	-	-
DQA1*0102	0.28	0.0221	-		Systemic Lupus Erythematosus risk	[4]
DQA1*0103	0.13	0.001	-	-	-	
DQB1*0201	0.12	0.0034	-	-	Addison's disease risk<br>Multiple Sclerosis risk (in LD with HLA-DRB1*03:01)<br>Sjogren's syndrome risk	[5]<br>[6]<br>[7]
DQB1*0301	0.28	0.0203	susceptibility to active TB<br>protective against active TB	[8]<br>[9]	-	-
DQB1*0501	0.07	0.0003	-	-	Sjogren's syndrome protection	[7]
DRB1*0102	5.92	0.0284	-	-	Rheumatoid Arthritis risk - shared epitope positive (QRRAA)	[10]
DRB1*1302	0.29	0.0452	susceptibility to active TB	[8]	Rheumatoid Arthritis protection - DERAA peptide allele	[11]

References

1. Axelsson-Robertson R, Ahmed RK, Weichold FF, Ehlers MM, Kock MM, Sizemore D, et al. Human Leukocyte Antigens A*3001 and A*3002 Show Distinct Peptide-Binding Patterns of the <i>Mycobacterium tuberculosis</i> Protein TB10.4: Consequences for Immune Recognition. Clinical and Vaccine Immunology. 2011;18(1):125-34. doi: doi:10.1128/CVI.00302-10.
2. Salie M, van der Merwe L, Möller M, Daya M, van der Spuy GD, van Helden PD, et al. Associations Between Human Leukocyte Antigen Class I Variants and the Mycobacterium tuberculosis Subtypes Causing Disease. The Journal of Infectious Diseases. 2013;209(2):216-23. doi: 10.1093/infdis/jit443.
3. Seedat F, James I, Loubser S, Waja Z, Mallal SA, Hoffmann C, et al. Human leukocyte antigen associations with protection against tuberculosis infection and disease in human immunodeficiency virus-1 infected individuals, despite household tuberculosis exposure and immune suppression. Tuberculosis. 2021;126:102023. doi: https://doi.org/10.1016/j.tube.2020.102023.
4. Morris DL, Taylor KE, Fernando MM, Nititham J, Alarcón-Riquelme ME, Barcellos LF, et al. Unraveling multiple MHC gene associations with systemic lupus erythematosus: model choice indicates a role for HLA alleles and non-HLA genes in Europeans. Am J Hum Genet. 2012;91(5):778-93. Epub 20121018. doi: 10.1016/j.ajhg.2012.08.026. PubMed PMID: 23084292; PubMed Central PMCID: PMCPMC3487133.

5. Ross I, Boulle A, Soule S, Levitt N, Pirie F, Karlsson A, et al. Autoimmunity predominates in a large South African cohort with Addison's disease of mainly European descent despite long-standing disease and is associated with HLA DQB*0201. Clin Endocrinol (Oxf). 2010;73(3):291-8. Epub 20100423. doi: 10.1111/j.1365-2265.2010.03807.x. PubMed PMID: 20455895.
6. Sawcer S, Hellenthal G, Pirinen M, Spencer CC, Patsopoulos NA, Moutsianas L, et al. Genetic risk and a primary role for cell-mediated immune mechanisms in multiple sclerosis. Nature. 2011;476(7359):214-9. Epub 20110810. doi: 10.1038/nature10251. PubMed PMID: 21833088; PubMed Central PMCID: PMCPMC3182531.
7. Cruz-Tapias P, Rojas-Villarraga A, Maier-Moore S, Anaya J-M. HLA and Sjögren's syndrome susceptibility. A meta-analysis of worldwide studies. Autoimmunity Reviews. 2012;11(4):281-7. doi: https://doi.org/10.1016/j.autrev.2011.10.002.
8. Lombard Z, Dalton D-L, Venter PA, Williams RC, Bornman L. Association of HLA-DR, -DQ, and Vitamin D Receptor Alleles and Haplotypes with Tuberculosis in the Venda of South Africa. Human Immunology. 2006;67(8):643-54. doi: https://doi.org/10.1016/j.humimm.2006.04.008.
9. Oliveira-Cortez A, Melo AC, Chaves VE, Condino-Neto A, Camargos P. Do HLA class II genes protect against pulmonary tuberculosis? A systematic review and meta-analysis. Eur J Clin Microbiol Infect Dis. 2016;35(10):1567-80. Epub 20160713. doi: 10.1007/s10096-016-2713-x. PubMed PMID: 27412154.
10. de Vries N, Renningen KS, Tilanus MG, Bouwens-Rambouts A, Segal R, Egeland T, et al. HLA-DR1 and rheumatoid arthntis in Israeli Jews: Sequencing reveals that DRB1* 0102 is the predominant HLA-DR1 subtype. Tissue Antigens. 1993;41(1):26-30.
11. Bettencourt A, Carvalho C, Leal B, Brás S, Lopes D, Martins da Silva A, et al. The Protective Role of HLA-DRB1(∗)13 in Autoimmune Diseases. J Immunol Res. 2015;2015:948723. Epub 20151029. doi: 10.1155/2015/948723. PubMed PMID: 26605347; PubMed Central PMCID: PMCPMC4641944.
