# Supplementary material for: The association of class II HLA alleles with tuberculosis-associated immune reconstitution inflammatory syndrome
Source: PLoS Pathog. 2025 Sep 19;21(9):e1013497. doi: 10.1371/journal.ppat.1013497 (PMC12510654; doi:10.1371/journal.ppat.1013497)
Supplement: S1 Text — (PDF) [file ppat.1013497.s010.pdf]

Supporting Information (SI)

Supplementary Materials and Methods

DNA isolation.

DNA was extracted from blood of 210 PredART participants [1] using the QIAamp DNA blood mini and midi kits (Qiagen), following the manufacturer's protocols. The NanoDrop 2000 spectrophotometer (Thermo Scientific) or BioDrop were used to measure DNA concentrations and purity. TE buffer was used as the reference sample (blank). Samples were stored at -20°C until HLA high resolution genotyping and other sequencing.

HLA Genotyping.

DNA extracted and purified from blood samples was sent to the institute for immunology and infectious diseases (IIID) at Murdoch University for HLA typing. HLA testing at IIID has been accredited by the American Society for Histocompatibility and Immunogenetics (ASHI) and the National Association of Testing Authorities (NATA). Specific HLA loci on the extracted DNA were PCR amplified using sample specific molecular indexed primers (MID-tagged) that amplify polymorphic exons from class I (A, B, C exons 2 and 3) and class II (DQ, exons 2 and 3; DRB and DPB1, exon 1) HLA genes. MID-tagged primers have been optimised to minimize allele dropouts and primer bias. Amplified DNA products from unique MID-tagged products were pooled in equimolar ratios and subjected to library preparation. Post QC and quantitation, the normalised libraries were then sequenced on the Illumina MiSeq platform using the MiSeq V3 600-cycle kit (2X300bp reads). Sequences were separated by MID tags and alleles called using an in-house accredited HLA allele caller software pipeline that minimises the influence of sequencing errors. Alleles were called using the latest IMGT HLA allele database as the allele reference library. Sample to report integrity was tracked and checked using proprietary and accredited Laboratory Information and Management System (LIMS) and HLA analyse reporting software that performs comprehensive allele balance and contamination checks on the final dataset

Full allelic KIR genotyping.

Uniquely indexed primers were designed to target KIR exons 3, 4 and 5, (D0, D1 and D2 Domain). Primer mixes were prepared containing 2-4 primers per amplicon to cover all KIR genes in a multiplexed assay with an amplicon size ~400 bp. The primers chosen have been adapted and refined from published literature [2]. Five separate PCR reactions were setup for each sample to cover all target exons. In a 96-well plate, there was 12.5µl volume with GoTaq DNA Polymerase (Promega) and the associated buffer system. As each sample was uniquely indexed during the PCR reactions, all amplicons from the PCR reactions were pooled using volumes appropriate to obtain balanced read coverage for each KIR exon. Each pool was then ligated with unique Illumina indexes and sequencing adapters ready for Illumina sequencing. The products were sequenced on the MiSeq using 600V3 chemistry. Post sequencing, quality filtered paired reads were demultiplexed based on unique molecular sequence for each sample and each set of overlapping paired reads were merged based on the Q30 scores leading to a single read with a median read length of up to 500bps spanning the full amplicon. Since these amplicons span across the full exon, the SNPs within the exon are phased. These reads were then aligned to a reference sequence containing all KIR genes using CLCbio genomics workbench. Following alignment, using an inhouse developed application All Class the reads were moved to the correct gene by comparing each mapped read to the reference gene dataset generated from IMGTKIR database; reads that did not map to any reference sequence data and reads mapping below the defined minimum cut off were discarded. Multi mapped reads were extracted and reassigned based on set defined rules based on KIR haplotypes and heterozygosity and final assignments were reported as igroups http://www.iiid.com.au/s/iiid_KIR_iGroups_v2100.xlsx. This entire workflow has been validated for precision, accuracy and specificity using the reference IWHG cell lines with known KIR alleles.

ERAP sequencing.

DNA from multiple samples were processed using massively parallel sequence-based typing where, 10 PCR primers pairs were utilised to amplify targeted regions which contain 17 SNPs for ERAP1 and 2 SNPs for ERAP2. Amplified DNA products from unique MID (molecular sample index) tagged products were pooled in equimolar ratios and subjected to library preparation. Post QC and quantitation

the normalised libraries were then sequenced on the Illumina MiSeq platform using the MiSeq V3 600-cycle kit (2X300bp reads). Post sequencing, sequences were separated by unique MID tags and aligned using CLCbio genomics workbench. Sequence visualising and analysis tool VGAS was then used for identification and reporting of the specific SNP variants and haplotypes.

References

1. Meintjes G, Stek C, Blumenthal L, Thienemann F, Schutz C, Buyze J, et al. Prednisone for the Prevention of Paradoxical Tuberculosis-Associated IRIS. New England Journal of Medicine. 2018;379(20):1915-25. doi: 10.1056/NEJMoa1800762. PubMed PMID: 30428290.

2. Lebedeva TV, Ohashi M, Zannelli G, Cullen R, Yu N. Comprehensive approach to high-resolution KIR typing. Human Immunology. 2007;68(9):789-96. doi: https://doi.org/10.1016/j.humimm.2007.07.002.
